# Remodeling of the tumor/tumor microenvironment ecosystem during KRAS G12C inhibitor clinical resistance in lung cancer

**DOI:** 10.1172/JCI156891

**Published:** 2022-02-15

**Authors:** Tadashi Manabe, Trever G. Bivona

**Affiliations:** 1 Department of Medicine,; 2 Department of Cellular and Molecular Pharmacology, and; 3 Helen Diller Family Comprehensive Cancer Center, UCSF, San Francisco, California, USA.

## Abstract

KRAS G12C inhibitors such as sotorasib and adagrasib are often effective in KRAS G12C–driven non–small cell lung cancer (NSCLC) patients. However, acquired resistance limits long-term patient survival. In this issue of the *JCI*, Tsai et al. present a comprehensive genetic analysis of multiple tumors with acquired sotorasib resistance obtained through an autopsy of a patient with KRAS G12C–mutant NSCLC. This analysis of pre- and posttreatment tumors uncovered cancer cell–intrinsic and –extrinsic features of resistance, including reactivation of KRAS-mediated signaling, reprogramming of metabolism, epithelial-mesenchymal transition, and tumor microenvironment changes. This elegant study demonstrates the multifaceted nature of KRAS G12C inhibitor clinical resistance and potential avenues to overcome resistance.

## KRAS inhibitors in lung cancer

Currently, 1 in 2 to 3 people will develop cancer, and among them, lung cancer remains the leading cause of cancer mortality. Substantial breakthrough discoveries, including the identification of lung cancer–specific oncogenic drivers (e.g., EGFR mutations, EML4-ALK fusion genes) and the development of molecular inhibitors of these pathogenic factors, have improved outcomes for patients with advanced-stage lung cancer. However, frequent oncogenic driver mutations that have remained undruggable until recently include activating mutations in the gene encoding the small GTPase KRAS. Hotspot mutations that predominately occur in codons 12 and 13 result in defective KRAS GTPase activity, thus enhancing the abundance of the active GTP-bound state in cells to promote cancer cell proliferation and growth via multiple downstream effector pathways such as the RAF/MEK/ERK (MAPK) pathway.

In recent years, a substantial advance occurred through the identification and development of covalent allosteric inhibitors especially targeting KRAS G12C (1–4), a mutation that comprises 41% of KRAS non–small cell lung cancer (NSCLC) (5). One drug, sotorasib (AMG510), inhibits reactivation by nucleotide exchange, thereby better confining KRAS to an inactive GDP-bound state (1). A subgroup of patients with NSCLC that underwent a phase I trial of this drug showed a 32.2% response rate and had 6.3 months of progression-free survival (6). Based on these results, sotorasib was granted accelerated approval by the US Food and Drug Administration in May of 2021 for adult patients with advanced KRAS G12C–mutant NSCLC who received at least one prior systemic therapy. While the development of KRAS inhibitors has been a tremendous success, resistance occurs in almost all patients and limits long-term patient survival. A better understanding of the mechanisms of resistance to KRAS inhibitors is paramount to maximize the potential of these emerging agents and improve clinical outcomes.

A variety of resistance mechanisms have been reported in preclinical studies of KRAS G12C inhibitors (2). Additionally, some mechanisms of clinical acquired resistance to KRAS G12C inhibitors have also been reported (6–8). One challenge that often arises in studies using clinical samples is ensuring ideal controls. For instance, pre- and posttreatment pairs and comparisons with normal tissue are desirable but often difficult to achieve in clinical practice.

## Mechanisms of clinical resistance to KRAS G12C inhibitors

**Figure 1:**
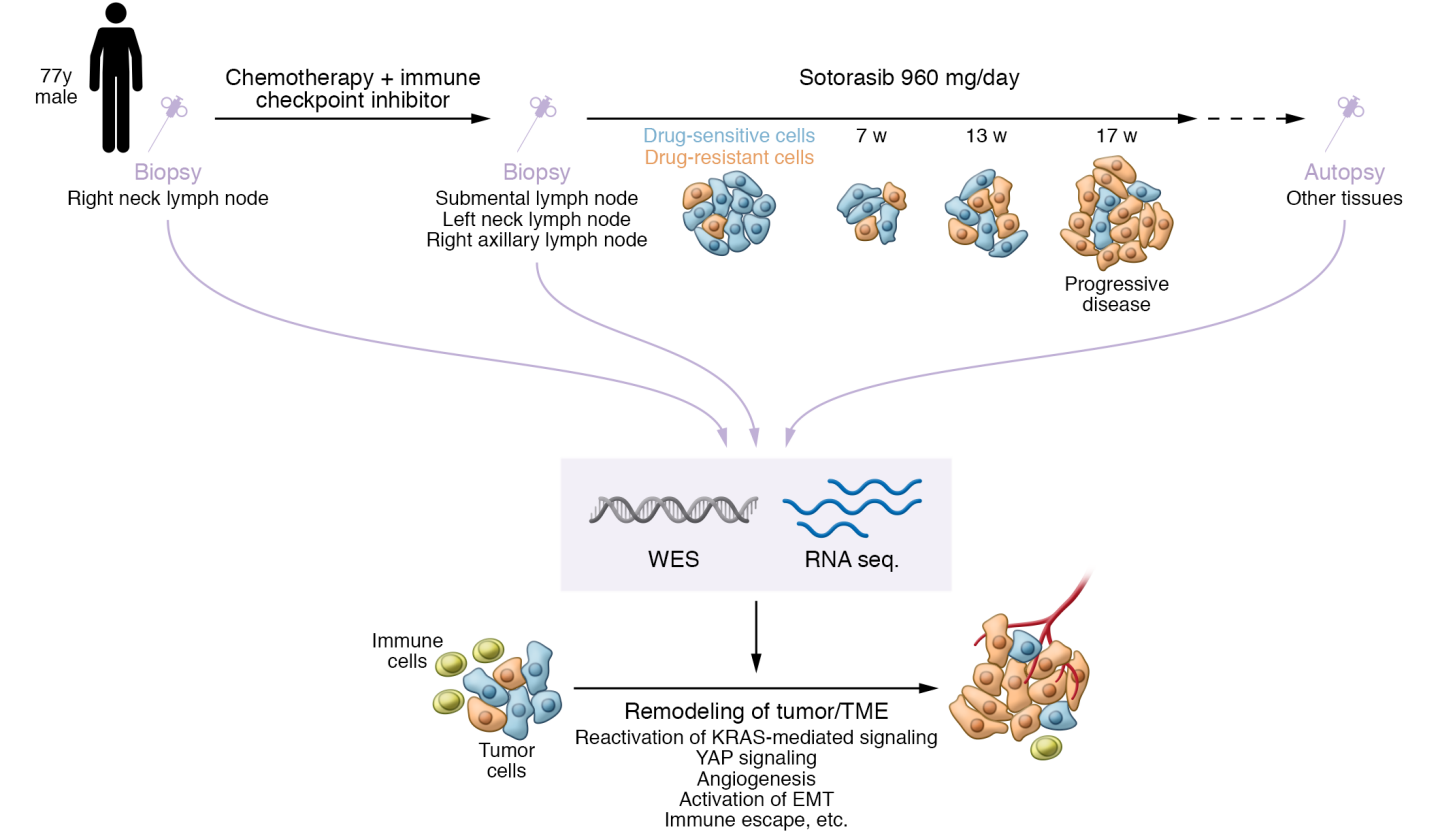
Schema showing the approach and main findings of Tsai et al. **(9).** Biopsy samples from a patient with KRAS G12C lung adenocarcinoma were taken before and after treatment with the KRAS G12C inhibitor, sotorasib. The patient was treated for 17 weeks and showed an initial response before developing acquired resistance. Whole-exome sequencing (WES) and RNA sequencing analyses of tumor and normal tissue samples showed KRAS-mediated signaling activation, YAP signaling, EMT activation, metabolic reprogramming, and TME changes that included coagulation, angiogenesis, and immune escape pathways.

In an elegant study published in this issue of the *JCI*, Tsai et al. (9) used paired pre- and post-sotorasib-treatment tissue samples and corresponding normal tissues to elucidate acquired mechanisms of clinical resistance to KRAS G12C inhibitors via comprehensive genetic analyses. The specimens used in this study were obtained from a patient with advanced-stage KRAS G12C lung adenocarcinoma treated with sotorasib for 17 weeks, with an initial response followed by acquired resistance (Figure 1). After succumbing to drug-resistant tumor progression, this patient underwent a rapid autopsy through which abundant tumor specimens and controls were obtained: 4 tumor tissues before treatment, 13 tumor tissues after therapy, and 8 nonadjacent normal tissues.

First, the authors performed transcriptome analysis of lymph node tumors and found that a total of 950 genes were differentially expressed after sotorasib treatment. Compared with pretreatment samples, activation of MAPK pathway, AKT, and mTOR signaling was present in almost all samples after sotorasib treatment. This result was consistent with previous reports describing that reactivation of MAPK and/or PI3K/AKT/mTOR pathways induce varying degrees of resistance (2, 10). However, unlike several previous reports investigating the mechanism of KRAS-inhibitor resistance using sequencing techniques (7, 8, 11), the authors did not find any additional mutations that reactivate KRAS or MAPK signaling. Whole-exome sequencing also showed a reduced KRAS G12C mutant allele frequency in most samples after treatment, suggesting that other factors maintain reactivation of the MAPK and/or PI3K/AKT/mTOR pathways. Activation of YAP signaling as a compensatory pathway supporting drug resistance was also present in the resistant tumors, consistent with preclinical studies (12).

In the 9 hallmark gene sets analyzed, 2 cell cycle gene sets (G_2_/M checkpoint and E2F target) were decreased, suggesting abnormal cell proliferation. The other 7 gene sets showed upregulation of a wide variety of pathways, including activation of hedgehog, NOTCH, WNT/β-catenin signaling, epithelial-mesenchymal transition (EMT), and tumor angiogenesis.

Phylogenetic analysis performed in Tsai et al. (9) revealed the process of inferred clonal evolution across metastatic lesions in different anatomic sites. Specifically, the pretreatment submental lymph node metastases had evidence of a common subclonal origin with the 6 posttreatment distant metastases, suggesting it seeded these distant posttreatment resistant tumors. Additional phylogenetic analysis of adjacent periportal lymph nodes showed a divergent inferred clonal origin. This result suggests that even adjacent metastatic lesions may arise from different clonal populations and have distinct resistance mechanisms, consistent with real-world clinical experience where treatment response can vary according to metastatic site.

Tsai et al. (9) also evaluated immunogenomic features of the tumors to understand how the immune microenvironment of tumors differs upon treatment exposure and resistance. The findings showed that multiple immune gene signatures associated with T and B cell function and activation were reduced in the posttreatment samples, resulting in an immunologically cold state.

## Conclusions and clinical implications

This important study by Tsai and colleagues illuminates the multifaceted mechanisms of acquired resistance to direct inhibition of KRAS G12C, including the reactivation of KRAS-mediated signaling, YAP signaling, the activation of EMT, metabolic reprogramming, and diverse changes in the tumor microenvironment (TME), including coagulation, angiogenesis, and immune escape pathways (ref. 9 and Figure 1). This study highlights mechanisms that warrant validation in other patients with KRAS G12C NSCLC and in preclinical models, as showing generalizability and causation are critical areas for future investigation. It is also important to define the extent to which the mechanisms of resistance to the different KRAS inhibitors, in clinical use or development, are shared or distinct. Overall, Tsai et al. (9) show the utility of obtaining and assessing clinical specimens as pre- and posttreatment pairs, comprehensively across the tumor/TME ecosystem, and characterizing both DNA and RNA levels, as well as normal tissue controls, to identify tumor-specific resistance alterations. Comprehensive tissue-based molecular analyses of tumors with clinical resistance to KRAS G12C inhibitors, coupled with liquid biopsy assessment and preclinical mechanistic studies, should yield a full evolutionary map by which tumors develop resistance. This evaluative process is essential to lay the groundwork for testing mechanism-based therapeutic strategies to thwart resistance and improve clinical outcomes for patients with KRAS G12C–driven NSCLC and potentially other KRAS-driven tumor types.

## Version 1 02/15/2022

Electronic publication
